# Development of a Technology-Based Intervention to Improve Help-Seeking in Distressed Non-Treatment-Seeking Young Adults With Common Mental Health Concerns

**DOI:** 10.7759/cureus.39108

**Published:** 2023-05-16

**Authors:** Prachi Sanghvi, Seema Mehrotra, Manoj Kumar Sharma

**Affiliations:** 1 Department of Clinical Psychology, National Institute of Mental Health and Neurosciences, Bengaluru, IND

**Keywords:** simple technology, mental health, young adults, intervention, help-seeking

## Abstract

Background

There is a dearth of interventions aimed at improving help-seeking for common mental health concerns among distressed young adults, particularly in the urban Indian context. Availability of cost-effective, targeted intervention for improving appropriate help-seeking can pave the way for reducing the treatment gap. This could prove especially beneficial in low-resource settings. This study describes the guiding principles, underlying theory, and development process of a simple technology-based help-seeking intervention for distressed non-treatment-seeking young adults.

Methods

Several models of professional help-seeking behavior were examined to ascertain a suitable theoretical framework for the development of the intervention to enable help-seeking among distressed non-treatment-seeking young adults. Pilot work was carried out before the development, along with content validation of the intervention by field experts.

Results

Help-seeking intervention was developed based on the preferences of young adults and literature review. Eight core intervention components and one optional component were developed, which were built on selected theoretical frameworks. These components have been postulated to enhance awareness of common mental health problems, the utility of self-help, and support of significant others, and to increase the skills to understand when it may be appropriate to step up to professional help-seeking.

Conclusion

Help-seeking interventions delivered beyond the traditional clinic and hospital setups prove useful as low-intensity interventions acting as gateways to seek mainstream mental health services. Further research will evaluate the feasibility, acceptability, and effectiveness of the intervention in reducing perceived barriers and enhancing inclination to seek professional help and help-seeking behavior among distressed non-treatment-seeking young adults.

## Introduction

As per the National Mental Health Survey 2015-16 [1], there is a high prevalence and wide treatment gap for common mental health problems, especially among urban young adults in India, leading to increased disease burden and disability. The lifetime prevalence for depressive disorders (8.23%) and neurotic and stress-related disorders (7.08%) was highest in urban metro areas, highlighting the burden of common mental disorders in urban India. However, low favorable attitudes and low inclination to seek professional help persist in young adults. Among those who do seek help, informal sources are preferred over professionals [2]. Systematic reviews identify cognitive, affective, and structural barriers that hinder professional help-seeking [3]. High preference for self-reliance, poor mental health literacy, stigma, need for privacy, a low perceived need for treatment, and self-perceived peer norms have emerged as key barriers to seeking professional help among young adults.

The widespread treatment gap for mental health problems has been attributed to demand- and supply-side barriers. The main demand-side barriers range from low perceived need, inadequate awareness, to certain sociocultural beliefs and stigma. In contrast, supply-side barriers include inadequate, unevenly disseminated, and inefficiently used resources [1]. In the past, efforts at reducing the treatment gap in mental health have focused largely on increasing access to mental healthcare through reducing supply-side barriers. However, the demand-side factors have received relatively less attention, perhaps due to their complex nature. Help-seeking interventions are interventions that address demand-side barriers. Thus, there is a need to develop effective and scalable interventions to improve help-seeking among distressed individuals keeping in view the sociocultural context.

Various interventions have been developed to reduce barriers to seeking professional help for mental health. These interventions are based on general health behavior models and those specific to help-seeking for mental health. Such interventions focus on changing help-seeking attitudes, inclinations, and behavior [4]. Interventions targeting behavior change have been most successful in altering health behavior [5]. These include mental health literacy and de-stigmatization programs, screening and linkage, contact with the intervention facilitator, and gatekeeper and peer training, among others [4]. Help-seeking interventions have been developed and deployed as universal and targeted interventions. Universal interventions have not shown consistent and significant change in help-seeking behavior [6]. This highlights the need to consider populations at risk or already suffering from mental health problems for early initiation and maintenance of treatment (targeted interventions).

Various intervention delivery modes have been explored in the literature, including face-to-face discussions, telephonic and online delivery modes, use of mobile phones, distribution of physical materials, and multimedia [7]. Use of simple technology such as online screening [8], text messaging [9], and incorporating simple visual interfaces [10] has been widely used in e-mental health interventions and services. Additionally, WhatsApp has been found to be an effective, safe, quick to use, and economic aid in the healthcare field [11]. It is a desired mode of contact for brief consultation, reference, and learning purposes among professionals and the public. WhatsApp-based programmatic interventions have also found a place in the literature [12]. Distressed young adults prefer technology for initial engagement with mental health services [13], which may increase their readiness to seek face-to-face mental health services. WhatsApp has been found to be the most preferred mode of communication, especially among young adults in the Indian context, for this purpose [14]. These findings led to the utilization of WhatsApp as the main form of intervention delivery, and via email as desired by the target population as part of the present intervention.

Although there is a voluminous literature on treatment gap, stigma, attitudes, and barriers toward help-seeking, there are fewer comprehensive studies on help-seeking interventions. Intervention studies with help-seeking variables often do not report the development process or describe it briefly. Without an explicit description of the underlying theoretical principles and the intervention development process, it becomes difficult to ascertain the potential reasons that may lead or not lead to the enhancement of help-seeking. A handful of intervention development frameworks for help-seeking have been elaborated in the literature, considering their usefulness in enhancing existing services and advancing the science of intervention development by testing it in diverse settings and populations [15].

The current study articulates the guiding principles, underlying theory, and development process of a simple technology-based multi-component help-seeking intervention for distressed non-treatment-seeking young adults. The goal of this intervention is to reduce barriers to help-seeking and enhance help-seeking inclination and behavior from mental health professionals for common mental health concerns. We intended to design the intervention in a way that lends itself to delivery using limited resources, minimal training, and large-scale deployment, especially in developing countries and low-resource settings.

## Materials and methods

Guiding principles for the development of help-seeking intervention

The intervention was developed based on the literature reviews [6,7] and the data from the exploratory study described elsewhere [16]. The following a priori principles were considered for relevant components deliverable using simple technology for the development of intervention.

The intervention content is expected to improve knowledge about common mental health concerns, self-awareness, enhance favorable attitudes toward mental health and persons seeking professional help for the same, and build or strengthen expectancies of recovery. The intervention content, format, and delivery mode would take into account the preferences of young adults in the urban Indian context, specifically those between 18 and 35 years of age with at least 12 years of formal education, with a self-report of the current experience of psychological distress for a minimum of two weeks, not seeking professional help for their present distress, having a working knowledge of the English language and access, and comfortable using the internet. Apart from the exploratory findings [16], literature reviews [6,7], and prior clinical experience of the authors, the intervention development was guided by theoretical frameworks described further. It was also planned to include multiple components for addressing a range of professional help-seeking-related factors, as the literature suggests the utility of multi-component interventions [7]. The intervention was expected to be deliverable as a low-intensity flexible intervention utilizing simple technology that is feasible to administer in a resource-limited setting. The potential for feasibility, ease of delivery by health providers with minimal training, and low-cost, large-scale deployment were important considerations for designing the intervention as it is meant to ultimately contribute to a reduction in the treatment gap.

The study was approved by the Human Ethics Committee, Behavioural Sciences Division, National Institute of Mental Health and Neurosciences (Approval no.: NO.NIMH/DO/IEC (BEH. Sc. DIV)/2019).

Planned intervention components

Based on the guiding principles, eight core components and one optional component were planned as part of the intervention (Table 1). Their focus is to enhance awareness about common mental health problems, utility of self-help, and support of significant others (SOs), and, at the same time, to develop and strengthen skills at understanding when it may be appropriate to step up from self-help and informal help to professional help-seeking. The planned intervention components, structure, and delivery modes correspond with the interventions described in various systematic and non-systematic reviews [4,7,17] and take into account the preferences of this sample obtained in the exploratory study [16]. These are elaborated in the subsequent section.

**Table 1:** Sequential list of help-seeking intervention components

Intervention component	Aim
Personalized feedback	To bring insight to one’s mental health status based on responses obtained on a standardized measure of distress and a few additional items in the screening
Recognition of common mental health concerns	To enhance the ability to identify common mental health concerns by understanding what a mental health concern is and its symptoms, and differentiating between sadness/depression and normal/excessive anxiety via an animated video clip
Overcoming barriers to consult a mental health professional	To reduce hesitation to seek professional help by addressing internal and external barriers in audio and poster format
Motivational interviewing	To enhance appropriate help-seeking by addressing individual barriers and motivating to seek help via single-session motivational interviewing and motivating video with prompts to facilitate self-reflection
Facilitating support mobilization from a nominated significant other for professional help-seeking (optional)	To improve confidence in seeking professional help by harnessing support from significant other through consenting for a single session between a significant person in one's life and the facilitator
Indirect social contact with mental health service consumers	To create a sense of relatedness through a narration of experiences of users of mental health services via a video clip
Socialization to the consultation process	To enhance knowledge on the process of professional help-seeking by educating about the need, benefits, types of professionals, and first-session expectations, and addressing confidentiality and good fit concerns in poster format
Experts’ view on mental health consultation	To create a sense of credibility and trust in mental health professionals by introducing the concept of mental wellness, comparing physical and mental health conditions, and introducing the role of professionals in treatment as explained by reputed mental health professionals in a brief video
Coordinated referral	To create easy access to appropriate mental health services by providing the intervention facilitator’s contact details at every point in the study and the option to seek referral-related guidance and support

Help-seeking intervention: utilization of theoretical frameworks

**Figure 1:**
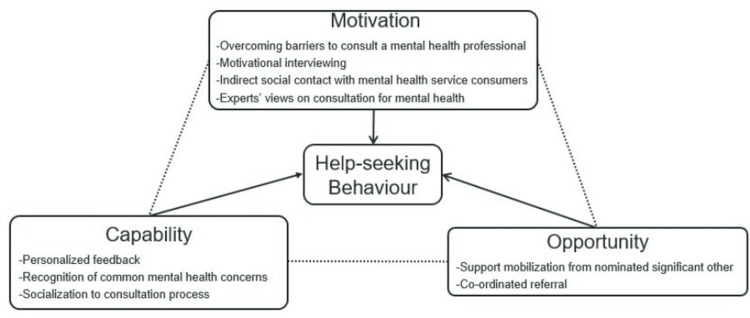
Linking the intervention components to the COM-B model

The intervention components reflect the theoretically driven framework of the behavior change wheel (BCW) [18], derived from a synthesis of 19 frameworks. BCW postulates three essential components, including capability, opportunity, and motivation, to bring about a behavior change (COM-B model). Capability is one’s physical and psychological ability to involve in a particular activity with the help of necessary knowledge and skills. Opportunities are those physical and social aspects that lie beyond an individual that makes the behavior occur. Motivation, in this model, is defined as those processes that guide behavior, e.g., goals, habits, emotional responses, and analytical decision-making. The COM-B model corresponds to the aim of behavior change in all the components of the present intervention and hence was considered the most important framework in the development process. Figure 1 shows the link between the study’s intervention components and the COM-B model. As the model suggests [18], the interaction of these three elements has been assumed to influence the change in help-seeking behavior.

BCW also incorporates intervention functions to address deficits in one or more of the aforementioned essential conditions. For the present study, these include education, persuasion, incentivization, training, environmental restructuring, modelling, and enablement (Table 2). The role of education is to increase knowledge about common mental health concerns and professional help-seeking. Persuasion and incentivization are used to create an expectation of positive feelings about addressing the current distress as part of the intervention by nudging them to seek appropriate help. The role of training is to impart skills in identifying common mental health concerns and learning when it may be appropriate to step up to professional help-seeking. Environmental restructuring in a social context is to mobilize support from SOs to facilitate help-seeking and to create easy access to mental health services. Modelling is aimed to provide an example for the target population to be inspired to take appropriate action by presenting the experiences of mental health service consumers. Lastly, enablement is meant to increase the means and reduce the barriers to increase capability and opportunity incorporated in all the components.

**Table 2:** Linking BCW intervention functions to intervention components 'X' indicates link between the study's intervention component and BCW intervention function. BCW, behavior change wheel

Intervention Components	BCW Intervention Functions						
	Education	Persuasion	Incentivisation	Training	Environmental Restructuring	Modelling	Enablement
Personalized feedback	X	X	X				X
Recognition of common mental health concerns	X	X	X	X			X
Overcoming barriers to consult a mental health professional	X	X	X	X			X
Motivational interviewing		X	X				X
Support mobilization from a nominated significant other		X	X		X		X
Indirect social contact with mental health service consumers		X	X			X	X
Socialization to the consultation process	X	X		X			X
Experts’ views on consultation for mental health	X	X	X				X
Coordinated referral							X

In addition, other relevant models developed explicitly to understand help-seeking among young adults were also used as guiding frameworks during the development. For instance, normalization and problematization of distress, and evaluation of consequences of help-seeking are the main reasons identified by Martínez-Hernáez et al. [19] for not seeking professional help among young adults, which have been addressed through the inclusion of components such as indirect social contact with mental health service consumers and motivational interviewing (MI). Biddle et al. [20] proposed distress to be on a continuum for young adults, where they try to normalize the distress and shift the threshold till it reaches a crisis stage. This was addressed in the intervention component of overcoming barriers to consult a mental health professional. Additionally, the assertion of the amount of gains and losses from help-seeking from Trusty et al.’s [21] behavioral economic model has been incorporated into the intervention through the reflective content on benefits of appropriate professional help-seeking versus potential impact of untreated mental health concerns included as part of the personalized feedback. Rickwood et al.’s [22] conceptualization of help-seeking as a social transaction has been considered in the intervention components comprising of support mobilization from SOs and providing coordinated referral to a mental health professional.

## Results

Intervention components: content and design

Personalized Feedback

The intervention is initiated with a feedback customized in accordance with a brief initial screening in a PDF format. The screening includes items to capture the level of psychological distress, coping strategies, help-seeking inclination, and barriers to seeking professional help. Personalized feedback is useful in helping individuals identify their mental health concerns and contribute to their decision to seek professional help [23]. It is one of the most effective ways of implementing behavioral change among various customizing processes [24]. The feedback template was validated during the exploratory study [16] as a precursor to the intervention development. The feedback is expected to provide insight into one’s current distress level. It also includes possible causes of distress and its possible impact on the functioning and usefulness of the coping strategies used to manage the distress. It throws light on the impact of an untreated mental health concern and benefits of addressing the same. Appropriate recommendations are included based on one’s distress level, along with the facilitator's contact details for clarifications and referral-related queries. Suggestions to seek professional help are embedded in the feedback in a way that encourages seeking professional consultation for a better understanding of the nature of the problem and subsequent guidance rather than implying the presence of a diagnosis and need for treatment.

Recognition of Common Mental Health Concerns

Information to increase recognition of common mental health concerns, including depression and anxiety, is planned subsequently to psycho-educate the target group and improve their mental health literacy. This component is next in the sequence since one of the primary reasons for not seeking professional help is the lack of recognition that one may be suffering from a mental health condition [3]. This is particularly important for the target population, given that they would be distressed and at risk of developing a mental health concern. This component includes an appealing animated video addressing the difference between sadness and depression and between normal and excessive anxiety. It speaks about what constitutes a mental health concern and prevalence of the same. The video also focuses on treatment options and the role of mental health professionals in treating the same.

Overcoming Barriers to Consult a Mental Health Professional

Increased mental health literacy alone may not necessarily lead to help-seeking behavior [4]. Hence, addressing the barriers is planned next to target the stigma, attitudinal, and instrumental barriers that delay professional help-seeking. This component involved two formats: audio followed by a poster. For the audio content, the authors selected the top five barriers by combining the findings obtained in the exploratory work [16] and individual rankings of all authors on top perceived barriers of young adults. Subsequently, the audio was recorded by volunteers in a dialogue form between a young adult and a professional. The remaining barriers were addressed in the poster.

Motivational Interviewing

As goal-directed MI enhances readiness to engage in treatment [17], a single-session phone call was sequenced next to address individual barriers and provide an opportunity to clarify any concerns related to professional help-seeking. Based on the four processes of MI [25], a template for the session is developed to be used with MI-consistent language in the session. After the session, a reflective animated video based on MI principles is sent to the individuals.

Facilitating Support Mobilization from a Nominated Significant Other

Encouragement from friends and family to seek support facilitates professional help-seeking. Specifically, in the Indian context, help-seeking is seen as a family-based decision-making process [6]. Thus, the optional component of facilitating support mobilization from a nominated significant other (NSO) via a single-session phone call is planned next and conducted as follows.

At the end of MI session, the individual is introduced to this component. The importance of support from significant others (SOs) during their times of distress and in consulting a mental health professional is discussed with them. Upon expression of interest, they are asked to nominate an SO they trust and are aware of their distress, at least to some extent. The target sample is explained that a general discussion would be held with the NSO to enhance their awareness of common mental health concerns, role of mental health professionals in addressing the same, and ways in which the NSO can support the target group and clarify any concerns they may have. The target sample is assured that no personal details will be revealed to the NSO unless they consent to the same. They are also assured that the NSO will not be asked to monitor them in any way or force them to consult a professional as the individual has complete autonomy to make that decision. Upon obtaining consent from the target sample, the NSO is contacted, and their consent is sought before initiating the session at a mutually convenient date and time.

A template has been developed to be used as a guide during this session. After the session, a flyer on supporting someone with a mental health concern is sent to them on their preferred communication mode, summarizing all the points discussed during the session.

Indirect Social Contact with Mental Health Service Consumers

Indirect social contact with mental health consumers has been found to significantly reduce stigma and improve help-seeking behavior [26]. Therefore, experience of individuals who sought professional help is the next sequenced component. It has been developed in a video format using the transcripts of the recorded interviews from the exploratory work of this intervention [16]. Volunteers recorded selected quotes from the transcripts, which were divided into the following sections: experience of distress, barriers in consulting a mental health professional and how they overcame those, and the impact of seeking professional help on their lives.

Socialization to Mental Health Consultation Process

One of the major barriers to not seek professional help is uncertainties about the process of service utilization. Apart from recognizing one’s mental health concern, the individual ought to know the availability of a range of mental health services and evidence-based treatments to obtain effective help [27]. Therefore, the next component planned is to introduce them to the mental health consultation process. This is developed in the poster form with seven sections, including when an individual should consult a mental health professional and benefits of the same, different types of mental health professionals and how one can reach out to them, expectations from the first session, and addressing confidentiality concerns and what the person can do in case they feel that the professional is not a good match for them.

Experts’ View on Consultation for Mental Health

Concern about the mental health professional's credibility is another reason that stops distressed young adults from seeking professional help [16]. Therefore, the last component to be presented is a video by mental health experts on the importance and need for mental health consultation and the role of professionals in the same. The video consists of reputed mental health professionals, including a clinical psychologist and a psychiatrist explaining mental wellness, comparison of help-seeking between physical and psychiatric conditions, impact of distress on well-being, availability of effective treatment options, and role of a mental health professional in addressing the same.

Coordinated Referral

It is planned to provide contact details to reach out to the intervention facilitator throughout the process to seek clarification or guidance for referral to a mental health professional. It has a substantial potential to encourage non-treatment-seeking individuals to consult a professional [13]. Contact details can be made available at the end of all components to connect with the facilitator at any point during the intervention. Alongside, a verified list of helpline numbers and contact details of mental health professionals in major cities and across the country can be kept available with the intervention facilitator for use as needed. In cases where the participants request assistance for consultation, a brief discussion can be held regarding their preferences. The facilitator can subsequently contact the potentially suitable professionals, and the referral can be discussed with consent, following which the professionals’ contact details may be shared with the distressed individuals.

Intervention format and delivery

The intervention is meant to be based on simple technology considering young adults' preferences and deliverable as a low-intensity intervention. It should have the potential for ease of delivery with minimal orientation and training and the potential for cost-effectiveness and large-scale deployment. In this context, simple technology meant the development and delivery of components that require basic and feasible formats such as audio, video, and image files to sustain engagement and delivered via preferred communication mediums such as WhatsApp and email for meaningful impact. Each component is delivered via Google form as an attachment to the target group’s preferred communication mode. This is intended to verify if the individual engaged with the components. Additionally, to confirm if the individuals sufficiently review the components, one to two questions on the related content are asked after presenting the component to examine their comprehension of the topic at hand.

Procedure

**Figure 2:**
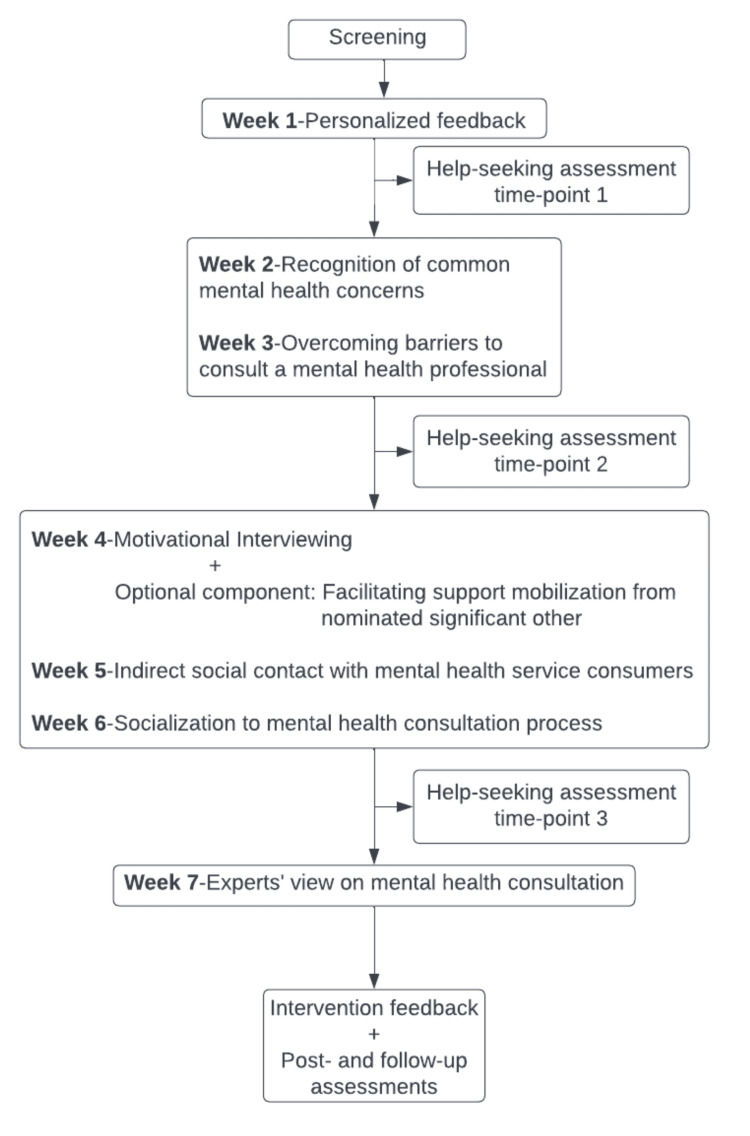
Structure of the help-seeking intervention

Figure 2 summarizes the intervention structure. The components are planned to be delivered in a spaced-out fashion. A range of 5-9 days with an average of seven days of the interval to deliver each component is decided depending on when the individual engages with the component. A maximum of two reminders are sent for each component in the interval period. Assessment of help-seeking inclination and behavior is carried out at three time-points to assess the change and as an indication of the outcome. If the individual reports seeking professional help at any point during the intervention, it is considered an end-point of the intervention for them. Depending on the strength of barriers and ambivalences, there are likely to be individual differences in the amount of time and engagement with the intervention components needed by each individual. Thus, it is expected that help-seeking outcomes would be achieved at variable points during the intervention period, and not all individuals might be required to engage in the entire intervention.

Content validation of the newly developed intervention

Following the development, the intervention was subjected to content validation by three mental health experts, including two clinical psychologists and one psychiatrist with a minimum of five years of professional experience. They were sought out due to their extensive experience in delivering expert clinical services, organizing programs aimed at raising awareness, reducing stigma, and promoting well-being for the public, especially young adults. The experts were individually asked to comment on the appropriateness of the content and structure of the intervention. A validation form comprising two sections was sent to the experts for evaluation. Ratings across three experts were averaged for each item across both sections. The first segment included 10 items focusing on intervention components and baseline assessment. The items assessed the appropriateness of the content concerning the target population. It was rated on a 4-point Likert scale from “not at all appropriate (1)” to “highly appropriate (4)” with an additional open-ended item for any comments. Each intervention element received an average score of 3.67 or more out of four, indicating high positive ratings for each component.

The second section consisted of five items to be rated by the experts on their overall feedback on the intervention. The items assessed the following parameters: a) the content adequately covered the goals of the intervention, b) appropriateness of the content to the proposed target population, c) the logical sequencing of the content in each component, d) the adequacy of the components to sustain interest, and e) clarity and ease of understanding of the components presented to the target population along with an open-ended item for any comments. Each item was to be rated on a 5-point Likert scale from “strongly disagree (1)” “strongly agree (5).” Each parameter received an average score of 4.67 or more out of 5, indicating high positive ratings. Suggestions to fine-tune the intervention by the evaluators were taken into consideration, and modifications were made accordingly, for example, the inclusion of baseline assessment of sources of emotional support in the target population, minor component formatting edits, and so on.

Thus, the newly developed help-seeking intervention was ready for pilot trial testing to examine its feasibility, acceptability, and effectiveness.

## Discussion

This study describes the underlying theory, guiding principles, and development process of a simple technology-based multi-component help-seeking intervention for distressed non-treatment-seeking young adults. Literature review underscored the need for this intervention in the target population in the Indian context [6,7]. Young people are highly unlikely to seek professional help, leading to short- and long-term functional impairment, diminished education and occupation opportunities, and possible comorbidities [28]. The reasons for not seeking professional help among young adults have been examined in various studies [16]. Indian literature on help-seeking points to under-utilization of mental health services [6]. Most studies have been conducted to explore pathways to care and barriers to professional help-seeking. Studies barely focus on non-treatment-seeking distressed individuals. This pointed to the need for developing intervention that encourage professional help-seeking for early detection and initiation of treatment.

Various behavior change interventions for help-seeking in mental health, including those with common mental disorders [4], among young adults have been developed and tested to reduce help-seeking barriers for self-identification of symptoms and facilitate early treatment. Majority of the studies across the globe are in the form of universal intervention targeting the general population [7]. They do not often consider barriers particularly relevant to a given segment of the population (e.g., high preference for self-reliance, privacy, flexibility, self-perceived peer norms, and so on, in young adults). Though these interventions have shown improved attitudes toward mental illness and help-seeking, these have not been found to be consistently associated with an increase in help-seeking behaviors. Although attempts have been made to increase help-seeking behavior among young adults, several studies have focused only on one or two components relevant to changing help-seeking patterns [7]. This led us to develop a targeted help-seeking intervention for distressed young adults. While education can be crucial in expanding knowledge and developing skills, it alone is usually insufficient to prompt behavioral changes [4]. In addition, individuals must possess the ability, motivation, and inclination to utilize their knowledge and skills. The present intervention was hence developed as a multi-component intervention targeting a range of relevant factors.

Intervention development necessitates guidance from theoretical models and frameworks. Various theories have been introduced in the last five decades to understand help-seeking in the context of health in general and mental health in particular. Interventions, including behavior change techniques, are most successful in altering health behavior [5] and have relevance for improving help-seeking. Behavior change interventions using BCW have been widely used in averting individuals from engaging in harmful or risky behaviors, taking part in protective behaviors, and endorsing effective self-management. BCW, which was chosen as the most relevant theoretical framework for developing the intervention in the present study, has been found to be effective in various behavior change interventions [29]. The facilitators of help-seeking for mental health identified in a handful of studies [3,6] include mental health literacy, positive past experiences, and social support and encouragement from SOs in the help-seeking process, and were utilized in the intervention developed.

There is growing global recognition of technology as one of the facilitators in bridging the treatment gap in mental health problems. Technology-based applications are being increasingly used as low-intensity interventions and aid to interventions guided by professionals in low-resource settings. Technology can be an influential mediator for help-seeking, particularly for young adults. The use of technology, especially by adolescents and young adults, has been on the rise to access mental health care. Young people use technology to retrieve information and guidance for mental health concerns. The internet has provided a space for young adults to feel in control, thereby giving them the confidence to discuss sensitive concerns [28]. These modes of delivering intervention have been effective in young adults due to increased confidence and ease in using technology and reduction in structural barriers and have a potential for wider reach [30]. The present help-seeking intervention is built on these observations and utilizes WhatsApp as the primary medium for delivery. The pilot testing of the intervention would help in gauging the extent to which the target population is receptive to and satisfied with the medium of delivery.

The newly developed help-seeking intervention aims to bridge the treatment gap by being a low-intensity, low-cost, flexible, and scalable intervention utilizing simple technology and feasible to administer by health professionals in a low-resource setting with minimal training. Availability of cost-effective, targeted intervention for improving appropriate help-seeking can pave the way for combining the same in future, with universal interventions directed at improving mental health literacy, informal support, and de-stigmatization in multi-pronged programs for improving help-seeking and reducing the treatment gap, especially in low- and middle-income countries. These interventions delivered beyond the conventional clinical settings may serve as useful low-intensity interventions acting as gateways to seek mainstream mental health services for individuals with unmet needs.

Way forward

A pilot trial is currently underway to evaluate the feasibility, acceptability, and effectiveness of this newly developed help-seeking intervention to reduce barriers and increase help-seeking inclination and behavior among distressed non-treatment-seeking young adults for common mental health concerns in the urban Indian context. The intervention is planned to be manualized for its standardized implementation.

## Conclusions

This study offers a comprehensive examination of the development process and design of a technology-based multi-component help-seeking intervention aimed at assisting distressed young adults. The development of this intervention was guided by a thorough analysis of empirical literature and relevant theoretical frameworks as well as an exploratory study.

The primary objective of this intervention is to reduce perceived barriers and enhance both the help-seeking inclination and help-seeking behavior among young adults with common mental health concerns. By addressing these barriers and promoting help-seeking, the intervention aims to bridge the gap in accessing appropriate treatment in low-resource settings. A detailed description of the development process is presented so as to explicate potential factors and mechanisms that may contribute to the effectiveness of the help-seeking intervention.

The developed help-seeking intervention shows promise as a feasible and scalable solution, particularly as it is designed to be a low-intensity intervention. The help-seeking intervention thus developed holds promise as a potentially feasible and scalable low-intensity intervention to reduce the treatment gap in low-resource settings and warrants further research.
